# MAC Project—Monitoring Anticoagulant Therapy Observational Study: Rationale and Protocol

**DOI:** 10.3389/fmed.2020.584459

**Published:** 2021-01-28

**Authors:** Giuseppe Camporese, Enrico Bernardi, Cristiano Bortoluzzi, Franco Noventa, Ngoc Vo Hong, Elena Callegari, Sabina Villalta, Chiara Tonello, Michela Nardin, Elena Campello, Luca Spiezia, Paolo Simioni

**Affiliations:** ^1^Unit of Angiology, Department of Cardiac, Thoracic and Vascular Sciences, University Hospital of Padua, Padua, Italy; ^2^Emergency Room, Department of Emergency and Accident Medicine, Conegliano Civic Hospital, Conegliano, Italy; ^3^Division of Internal Medicine, Department of Internal Medicine, Venice Cìvic Hospital, Venice, Italy; ^4^QUOVADIS Association & Department of Molecular Medicine, University Hospital of Padua, Padua, Italy; ^5^Division of Internal Medicine, Department of Internal Medicine, Treviso Civic Hospital, Treviso, Italy; ^6^General Medicine Unit & Thrombotic, and Haemorrhagic Disorders Unit, Department of Internal Medicine, University Hospital of Padua, Padua, Italy

**Keywords:** anticoagulants, direct oral anticoagulant, monitoring, deep vein thrombosis, pulmonary embolism

## Abstract

Real-life studies complement data from registrative trials. Because of the delayed registration of direct oral anticoagulants in Italy, scarce real-life data on such treatments is available for the Italian population. The aim of the MAC project is to collect real-life clinical information in unselected patients given oral anticoagulants for venous thromboembolism, during a 5-year follow-up period. This is a prospective-cohort, multi-center, observational study performed in four Italian centers. The estimated samples size is 4,000 patients. The efficacy outcomes are: incidence of symptomatic recurrent venous thromboembolism and of post-thrombotic syndrome. The safety outcomes are: incidence of major bleeding, clinically relevant non-major bleeding, minor bleeding, serious adverse events, and mortality. The MAC project has the potential to improve our understanding of the epidemiology and of the therapeutic strategies adopted in Italian patients with venous thromboembolism.

**Clinical Trial Registration**: WWW.ClinicalTrials.Gov, identifier: NCT0432939.

## Introduction

Anticoagulation, either administered via the parenteral (heparins and fondaparinux), or the oral route [vitamin K antagonists (VKAs)], represents the mainstay for prevention and treatment of venous thromboembolism (VTE), including deep-vein thrombosis (DVT), pulmonary embolism (PE), and superficial-vein thrombosis (SVT), since more than half a century. In recent years, a new class of anticoagulant drugs targeting the coagulation factors Xa and IIa, the so-called “direct” oral anticoagulants (DOACs), were developed and marketed. Currently, due to their comparable efficacy, higher safety, and easiness of use, DOACs have largely replaced VKAs in VTE treatment (1, 2). Besides landmark registrative trials, DOACs have been tested in numerous post-marketing studies, that substantially confirmed the generalizability and reproducibility of the the registrative trials' results, in which much broader selection criteria were applied (3–12).

Due to the late marketing of DOACs in Italy, scarce “real-life” data on the use of these drugs for the management of VTE is currently available for the Italian population. Thus, it is likely that a prospective data collection carried out in unselected consecutive patients with VTE could provide valuable information about the specific Italian context, accounting for potential differences linked to the genetic array or to the typical Italian diet/lifestyle. Furthermore, such a study might shed light on particular groups of patients, usually excluded or poorly represented in registrative studies, such as those with cancer, renal failure, low or high body mass index, transplant recipients, or on polytherapy (13).

To our knowledge, only one prospective observational registry (START2 Registry) recruiting unselected patients with VTE (14, 15) treated with anticoagulants, irrespective of the clinical indication, and of the class of anticoagulants (parenteral, VKAs, DOACs), is currently ongoing in Italy.

Thus, we embarked in a cohort study, designed to evaluate in the “real-word” setting the outcome of unselected Italian patients with VTE treated with anticoagulants, which will be followed-up for up to 5 years.

## Materials

### Design

The MAC Project is a multi-center, observational, investigator-initiated, cohort study. Specifically, we will gather information on clinical outcomes and on adverse events occurring to unselected patients with VTE treated with anticoagulants, with a specific focus on DOACs. The study protocol has been approved by the Institutional Review Board of each participating center. The MAC Project takes place in Italy.

### Population

**Figure 1:**
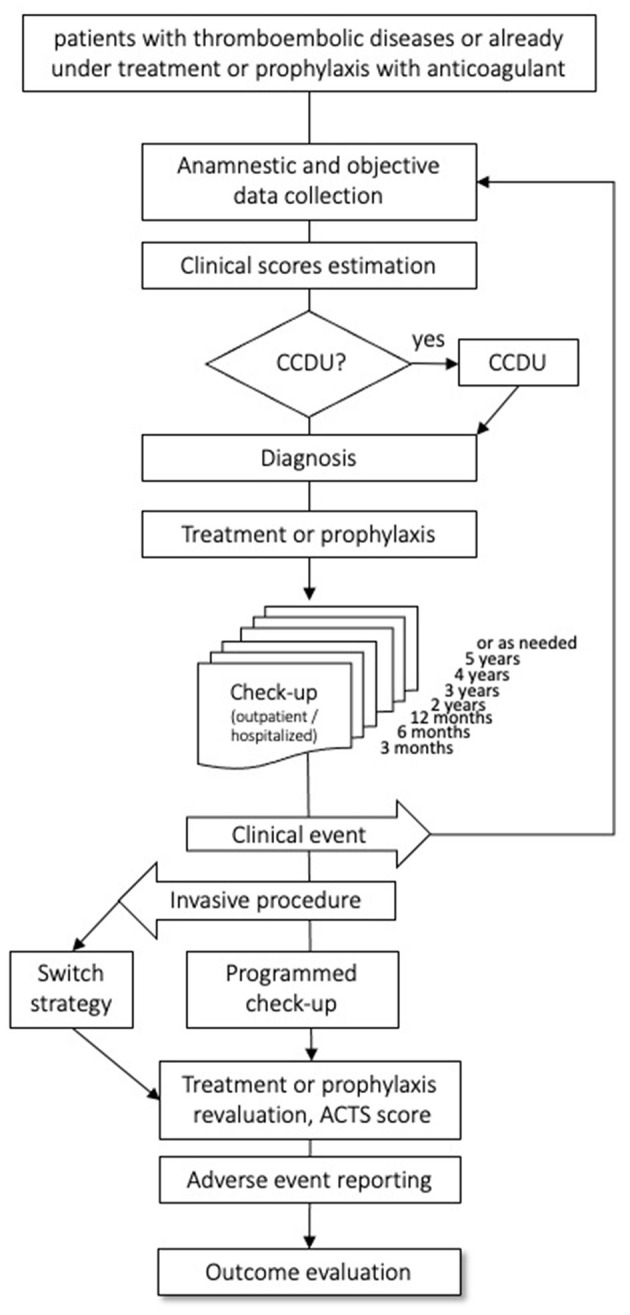
Flow of the patients through the observation period. CCDU, Color coded Doppler Ultrasound; ACTS, Anti-Clot Treatment Scale.

Subjects of both sexes, aged 18 years or older with objectively diagnosed VTE, irrespective of the index event, of the intended treatment duration, and of the type of anticoagulant (parenteral or oral) treatment, are eligible for the study. Patients already on anticoagulants for VTE are also eligible, provided that the time elapsed between the index event and the enrollment date is shorter than 1 year, and that complete information on the index event and on the course of anticoagulation before inclusion can be gathered retrospectively. There are no exclusion criteria, except for life expectancy <6 months, refusal to sign the informed consent form, and to attend the planned follow-up visit. All included patients will be followed-up prospectively for the first year after the index event, with scheduled medical visits at 3, 6, and 12 months, and thereafter annually for up to 5 years, as long as they are anticoagulated. In order to minimize the number of lost-to-follow-up, we will try to contact all patients not attending the scheduled control visit by phone or by mail. The patient flow is shown in Figure 1. Data on the patients' health statuses will be recorded at each follow-up visit, including the occurrence of safety or efficacy outcomes, or of adverse events, along with information on their management and outcome. Furthermore, we will document rehospitalizations, switch or withdrawal of anticoagulation, and occurrence of new diseases or treatments. In the case of a suspected event, as detailed in the patients' information sheets, patients are required to promptly contact the enrolling center, which is committed to organize a control visit within a short time frame. Patients enrolled after the index event will first undergo a run-in visit; subsequently, they will join the intended follow-up schedule.

Currently, Some 600 Patients Have Been Entered in the Database.

### Sample Size

According to recent literature, the expected frequency of serious adverse events in anticoagulated patients is 1.7%, that of major bleeding being 2.2% at 60 days (16). Based on these data, we calculated that by recruiting at least 4,000 subjects, the 95% confidence interval around the point estimate of serious events would be 0.4%, the respective figure for major bleeding being 0.5%.

Sample size was calculated by nQuery Advisor v.8 (Statsol LTD, Cork, Ireland) (17).

### Outcomes

As efficacy outcomes we chose: the incidence of symptomatic recurrent VTE; and that of post-thrombotic syndrome. PE will be adjudicated by abnormal Computed Tomography Pulmonary Angiogram (CTPA); by abnormal perfusion lung scan plus normal chest X-ray; by the finding of DVT on ultrasonography in the presence of typical symptoms; or by autopsy. DVT and SVT will be ruled-in on the basis of whole-leg color-coded ultrasonography, according to a standardized protocol, described elsewhere, using vein incompressibility as the sole diagnostic criterion (18). Post-thrombotic syndrome will be diagnosed on clinical grounds, using a validated scale (the Villalta Score) described elsewhere (19).

As safety outcomes, we chose the incidence of: major bleeding, as defined by the International Society on Thrombosis and Haemostasis (ISTH) criteria; clinically relevant non-major bleeding; minor bleeding; serious adverse events; and mortality, either VTE-related, cardiovascular, or all-cause (20). Additionally, we will also evaluate treatment adherence and patients' satisfaction, using a validated score (Anti-Clot Treatment Scale) (21).

### Statistical Analysis

Outcome analysis will be performed on all subjects enrolled in the study. In the case of multiple outcomes occurring to one patient, only the first event will be counted for the analysis. No pre-specified subgroup analysis is planned. All variables will be first analyzed descriptively; that is, categorical variables by means of frequency tables, and continuous variables by sample statistics, such as average, median, standard deviation, minimum and maximum value, and so on. Subsequently, putative risk factors for the specified outcomes, including obesity, varicose veins, cancer, standing or prolonged immobilization, recent surgery, severe infections, previous VTE, thrombophilia, chronic heart failure NYHA III-IV, and use of contraceptives, will be entered in the univariate analysis. All variables included in the model will be tested for the assumption of risks proportionality with the usual graphic method. The subset of variables significantly associated with the occurrence of safety and efficacy endpoints will be selected by the Wald method, employing a “forward stepwise” approach. Those variables found to be significantly associated with the endpoints at the univariate analysis will be entered in a Cox regression model for proportional risks, using age at the start of follow-up, gender, type of anticoagulant, and compliance to treatment, as covariates. Both unadjusted and adjusted cumulative proportions of event-free subjects during follow-up will be described by the Kaplan–Meier method. Differences between various types of treatment will be tested by the log-rank test. Subjects completing an uneventful follow-up, or dropping-out without experiencing events, will be censored, participating in the risk estimate with the available observation period. The results of the study will be reported in an integrated statistical and clinical report, compliant with the GCP-ICH guidelines.

### Data Collection

Data will be recorded on an “Electronic Data Capture” (EDC) system, based on the “Research Electronic Data Capture” online platform (REDCap, produced and distributed by Vanderbilt University and “REDCap Consortium”), in compliance with article n. 13 of the GDPR (EU 2016/769) (22). The MAC study e-CRF outline is available online (https://quovadis-ass.it/mac/eCRF.pdf).

Personal data of enrolled subjects will be treated with the maximum confidentiality, according to the terms of the Italian law (D.L. 211/2003 and subsequent amendments and additions) and GDPR [Regulation (EU) 2016/679 of the European Parliament and of the Council of 27 April 2016]. In particular, personal data of the subjects will be known only to the principal investigator and his collaborators who follow the patient and collect the treatment consent. Each enrolled subject will be distinguished only by a unique numeric identifier. The use and maintenance of the REDCap platform are guaranteed by an administrator who manages the user's privileges with a flexible and granular authorization system. REDCap applies the permissions granted to each user who connects via a web browser and the protocol with SSL encryption. REDCap allows a complete “audit” of the procedures performed by each user, by logging all operations on the data, including viewing and exporting. The operational control log (log) stores the date, time, and the user performing the operation, allowing a complete overhaul and remote monitoring of the clinical study if necessary. All users accessing the EDC platform (the investigators, the members of their staff, and the staff of the Promoter) must complete a training event, in order to increase the reliability, quality, and integrity of the data recorded in the EDC platform. REDCap implementations allow compliance with the most common industry standards and EMA requirements (GCP, Privacy-IT: D.L. 211/2003 and subsequent amendments and additions, GDPR and FDA (21-CFR2-Part 11).

### Centers and Investigators

Currently, the study is ongoing in four clinical centers (the General Medicine ward and Thrombotic and Haemorrhagic Disorders Unit at the Padova University Hospital; the Department of Emergency and Accident Medicine at the Conegliano, Vittorio Veneto, and Treviso Hospitals; the Department of Internal Medicine at the Venice Hospital; and the Department of Internal Medicine at the Treviso Hospital). All investigators are experienced in the management of patients with VTE.

### Coordinator

The study is coordinated by QUOVADIS, a recognized non-profit association, based in Padua, dedicated to scientific research in the field of diagnosis, prevention, and treatment of cardiovascular disease. All treatment options, including the choice of, the duration, and any dosage variations of anticoagulant treatment will be at the discretion and under the responsibility of both the attending physician and the patient, according to good clinical practice.

The study drugs will not be provided by a sponsor, and will be prescribed according to current standards of care and regulations.

## Conclusions

The MAC project is an ongoing registry, designed to provide insights into the clinical management and related outcomes in unselected patients with VTE treated with anticoagulants.

## Ethics Statement

The studies involving human participants were reviewed and approved by Ethics Committee of the University Hospital of Padua, Padua, Italy. The patients/participants provided their written informed consent to participate in this study.

## Author Contributions

GC, EB, CB, and FN had the idea for and designed the study. GC, EB, NH, EC, CT, MN, SV, EC, and LS will obtain and upload data. GC, EB, CB, and FN will analyze and interpret data. GC, ECal, EB, and FN will write the manuscript and finalize it. SV and PS will revise the final manuscript. All authors contributed to the article and approved the submitted version.

## Conflict of Interest

The authors declare that the research was conducted in the absence of any commercial or financial relationships that could be construed as a potential conflict of interest.
